# Ultrasound and microbubble-mediated delivery of miR-424-5p has a therapeutic effect in preeclampsia

**DOI:** 10.1186/s12575-023-00191-5

**Published:** 2023-02-15

**Authors:** Xudong Wang, Yan Wu, Qinliang Sun, Zhonghui Jiang, Guoying Che, Yangyang Tao, Jiawei Tian

**Affiliations:** Department of Ultrasound, the Second Affiliated Hospital of Harbin Medical University, No. 246, Xuefu Road, Nangang District, Harbin, Heilongjiang 150001 P.R. China

**Keywords:** miR-424-5p, Ultrasound and microbubble mediated gene delivery, AOC1, Preeclampsia, Trophoblast cells, Invasion, Migration

## Abstract

**Objective:**

To determine the influence of ultrasound/microbubble-mediated miR-424-5p delivery on trophoblast cells and the underlying mechanism.

**Methods:**

Blood pressure and 24-h proteinuria of patients with preeclampsia (PE) were measured as well as the levels of miR-424-5p and amine oxidase copper containing 1 (AOC1) in placental tissues. HTR-8/Svneo and TEV-1 cells were subjected to cell transfection or ultrasonic microbubble transfection for determination of the expression of miR-424-5p, AOC1, β-catenin and c-Myc as well as cell proliferation, apoptosis, migration and invasiveness. The concentrations of placental growth factor (PLGF), human chorionic gonadotropin (β-hCG) and tumor necrosis factor-α (TNF-α) were measured in HTR-8/Svneo and TEV-1 cells. RNA immunoprecipitation (RIP) and dual luciferase reporter assay detected the binding of miR-424-5p to AOC1. A PE mouse model was induced by subcutaneous injection of L-NAME, where the influence of ultrasound/microbubble-mediated miR-424-5p delivery was evaluated.

**Results:**

miR-424-5p was downregulated while AOC1 was upregulated in the placental tissues from PE patients. Overexpression of miR-424-5p activated Wnt/β-catenin signaling pathway and promoted the proliferation of HTR-8/Svneo and TEV-1 cells as well as enhanced the migratory and invasive behaviors. AOC1 overexpression partly eliminated the effects of miR-424-5p on HTR-8/Svneo and TEV-1 cells. Ultrasound and microbubble mediated gene delivery enhanced the transfection efficiency of miR-424-5p and further promoted the effects of miR-424-5p in trophoblast cells. Ultrasound/microbubble-mediated miR-424-5p delivery alleviated experimental PE in mice.

**Conclusion:**

Ultrasound and microbubble-mediated miR-424-5p delivery targets AOC1 and activates Wnt/β-catenin signaling pathway, thus promoting the aggressive phenotype of trophoblast cells, which indicating that miR-424-5p/AOC1 axis might be involved with PE pathogenesis.

## Introduction

Preeclampsia (PE) is a polygenic disorder featured by hypertension and proteinuria, which affects 2 ~ 8% pregnant women and threatens maternal and neonatal health and survival [1, 2]. The initiation of PE might be induced by multiple factors including insufficient acquisition of trophoblast cell invasion, abnormal endothelial function, oxygen dysregulation, and inflammation [3]. To improve the perinatal outcomes in PE patients, maternal factors combined with biomarkers are applied to predict PE, such as antiangiogenic factors and angiogenic placental growth factors (PLGF) [4]. Despite huge steps forward in the development of clinical care and extensive research, delivery is still the only effective method of managing maternal symptoms caused by PE [5]. Intriguingly, mounting evidence proves that impaired invasive and migratory capacities of trophoblast cells lead to severe pathological events of PE [6, 7]. Therefore, an increasing number of investigations focus on the molecular mechanisms implicating in trophoblast cell invasion and migration, which would be helpful for finding novel approaches and directions of PE treatment.

MicroRNAs (miRNAs) are small endogenous noncoding RNAs with 21 ~ 25 nucleotides in length that could regulate the expression of target genes through binding the 3’untranslated region (UTR) of downstream mRNA by translational suppression or degradation [8]. A number of miRNAs have been reported to regulate multiple biological processes of trophoblast cells, such as cell differentiation [9], invasion [10], proliferation and migration [11]. miR-424-5p has been demonstrated to restrain the migratory and invasive capacities of tumor cells in breast cancer [12]. However, in laryngeal squamous cell carcinoma, miR-424-5p exhibits a promotive role in cancer cell invasion and migration [13]. Moreover, miR-424 is uniquely downregulated in primary human trophoblast cells under hypoxic condition [14]. These findings noted that miR-424-5p might have an important role in the migratory and invasive capacities of trophoblast cells, whereas low circulation half-life of naked synthetic small RNAs in bloodstream is a big obstacle for using miRNAs as therapeutic tools [15]. Hence, an effective and safe method is required to stably carry a certain gene into targeted cells. Ultrasound-targeted microbubble destruction is a method for local drug or nucleic acid delivery to a target site by using microbubbles as vectors [16]. This method has been widely documented as a promising cancer therapeutic approach due to its satisfactory efficiency in delivery of a target gene to a local site [17]. Therefore, we speculated that ultrasonic microbubbles could carry miR-424-5p to trophoblast cells and affect cell migratory and invasive capacities.

Amine oxidase copper containing 1 (AOC1) is a human diamine oxidase involved in the extracellular degradation of histamine, with its expression mainly in the placenta, kidney, and intestine [18]. Histamine contributes to embryo–uterine interactions due to its vasoactive, differentiation, and growth-promoting properties, while AOC1 is produced in abundance by the placenta to prevent excessive entry of bioactive histamine from the placenta into the maternal or fetal circulation [19]. The balance between histamine and AOC1 seems to be crucial for an untroubled course of pregnancy. Therefore, we intended to further determine whether miR-424-5p regulates AOC1 expression during PE.

The goal of this investigation is to elucidate the function and mechanism of miR-424-5p in trophoblast cell migration and invasion and to illuminate whether ultrasound and microbubble mediated miR-424-5p delivery could stably transfect miR-424-5p into trophoblast cells. This study may provide a feasible approach and a promising target for increasing the aggressive phenotype of trophoblast cells, which could contribute to PE treatment.

## Materials and methods

### Ethical statement

Written informed consent form was acquired from patients or their families before collection and use of placental tissues in this study. All experiments have been allowed by the ethical committee of the Second Affiliated Hospital of Harbin Medical University and were carried out in strict accordance with the ethical principles for human subject research in Declaration of Helsinki.

### Bioinformatics analysis

GSE143953 dataset was recruited from GEO database (http://www.ncbi.nlm.nih.gov/geo), which included the mRNA profile of placental tissues from 4 patients with PE and from 4 normal pregnant women. Volcano plot of gene expression profile was described, in which the differentially expressed genes were highlighted. Log|FC|> 2 and *P* < 0.05 were set as filter criteria.

### Clinical samples

PE patients in early stage (*n* = 15) and normal pregnant women (*n* = 15) at the Second Affiliated Hospital of Harbin Medical University were enrolled in this study. PE patients recruited were diagnosed by at least two deputy chief physicians or chief physicians of the obstetrics and gynecology department. Include criteria were as follows: gestational age of 20 ~ 34 weeks; no specific medication during the pregnancy; the subjects were informed and agree to participate in this study; pregnant women underwent caesarean section. Pregnant women with one of the following conditions were excluded: multiple pregnancy; fetal chromosomal or structural abnormalities; chronic hypertension plus PE during pregnancy or pregnant woman with hypertension; pregnant woman with liver and kidney disease, cardiopathy or endocrine system disease. After caesarean section, placental tissues (1 cm^3^) were collected from maternal placentae within 10 min after delivery of placenta. The tissues were washed with normal saline, dried with sterile gauzes and preserved in liquid nitrogen. All recruited patients were subjected to blood pressure measurement for three times at different days, and the average value of these three independent data was regarded as the blood pressure. Urine of enrolled patients was collected in 24 h to measure the urine volume and the 24-h proteinuria.

### Cell culture

Human trophoblast cells (HTR-8/Svneo and TEV-1) and human embryonic kidney cells (HEK293T) acquired from American Type Culture collection (ATCC, Manassas, Virginia, USA) were maintained in DMEM/F12 (Gibco, Grand Island, NY, USA) containing 10% fetal bovine serum (FBS) and DMEM (Gibco, Grand Island, NY, USA) containing 10% FBS and 1% penicillin/streptomycin, respectively. Cell culture was carried out at 37℃ with 5% CO_2_.

### Cell transfections

miR-424-5p mimic (50 nM), vectors with AOC1 overexpression (2 μg), and the corresponding negative controls (mimic NC and pcDNA3.1) were synthesized by Shanghai GenePharma Co.,Ltd (Shanghai, China) and transfected into cells as the instructions on Lipofectamine 2000 reagent (Invitrogen, Carlsbad, CA, USA). Following measurements were carried out 48 h after the transfections.

### 5-Ethynyl-2’-deoxyuridine (EdU)

An EdU labeling/detection kit (Ribobio, Guangzhou, China) was used to examine cell proliferation. HTR-8/Svneo and TEV-1 cells in logarithmic phase were pipetted into 96 well plates at a density of 10^5^ cells/well. Culture medium was used to dilute EdU solution at 1000:1 to prepare culture solution containing 50 μM EdU. The HTR-8/Svneo and TEV-1 cells in each well were cultured with 100 μl EdU for 2 h. After the culture solution was discarded, the cells were washed with PBS once or twice for 5 min each time. Then, the cells in each well were fixed by 4% paraformaldehyde in 50 μl PBS for 30 min, cultured with 50 μl 2 mg/ml glycine for 5 min and washed in 100 μl PBS for 5 min. Afterwards, 100 μl 0.5% TritonX-100 was cultured with the cells for 10 min and then washed off by PBS for 5 min. Prepared 100 μl 1 × Apollo® solution was added onto the plate and cultured at room temperature away from light. Thirty minutes later, the 1 × Apollo® solution was removed and the cells were washed by 100 μl 0.5% TritonX-100 twice or three times for 10 min each time. Then, TritonX-100 solution was pipetted off and the cells were successively washed in 100 μl formaldehyde (once or twice, 5 min each time) and in PBS (once, 5 min). The cells were cultured with 100 μl 1 × Hoechst 33,342 solution in dark at room temperature for 30 min and washed by 100 μl PBS once to three times before being observed by a fluorescence microscope.

### Flow cytometry

Density of HTR-8/Svneo and TEV-1 cells in each group was adjusted to 10^5^ cells/ml, and 3 ml cell suspension was pipetted into a 10 ml centrifuge tube and centrifuged for 5 min at 500 rpm. After culture medium was removed, the cells were washed with PBS and centrifuged at 500 rpm for 5 min. Then, the supernatant was discarded and the cells were resuspended in 100 μl binding buffer. Following 5 μl Annexin V-FITC and 5 μl PI were added for culture for 5 min at room temperature in dark, the fluorescence intensity of FITC and PI was visualized by flow cytometry to measure cell apoptosis.

### Scratch assay

Transfected HTR-8/Svneo and TEV-1 cells were seeded onto six well plates at a density of 1 × 10^6^ cells/well in triplicate and then cultured at 37℃ with 5% CO_2_ for 24 h until monolayer cells covered the whole well. A sterile 200 μl pipette tip was used to scratch the monolayer cells. Afterwards, the cells were washed by PBS and further cultured for 48 h at 37℃ with 5% CO_2_. Gap of the scratch was observed and recorded by a microscope at the 0^th^ h and the 48^th^ h. Migration rate of trophoblast cells was measured based on the changes of the gap. Migration rate = (gap of the scratch at 0 h – gap of the scratch at 48 h)/gap of the scratch at 0 h.

### Transwell invasion assay

Transfected HTR-8/Svneo and TEV-1 cells were resuspended in serum-free culture medium and the density of the cells was adjusted. Then, 100 μl cells (1 × 10^5^ cells) were pipetted onto matrigel-coated transwell chamber (354,480, Corning, NY, USA). The basolateral chamber was covered with 600 μl culture medium containing 10% FBS. After cell culture for 24 h at 37℃, the transwell chamber was collected and the excess culture medium was removed. Non-invasive cells in the chamber were scraped off by wet cotton swabs. Cells in the chambers were fixed by 4% paraformaldehyde for 30 min, stained with Giemsa dye for 20 min and counted under a microscope.

### ELISA

Levels of PLGF, human chorionic gonadotropin (β-hCG) and tumor necrosis factor-α (TNF-α) in supernatant of HTR-8/Svneo and TEV-1 cell culture medium were quantified as described in the instructions of ELISA kit (R&D Systems, Minneapolis, MN, USA).

### RNA immunoprecipitation (RIP)

HTR-8/Svneo and TEV-1 cells were collected, washed with PBS twice and centrifuged at 1,500 rpm for 5 min. Afterwards, the cells were mixed with an equal volume of RIP lysis buffer. Magnetic beads were resuspended in 100 μl RIP Wash Buffer and incubated with 5 μg anti-Ago2 antibody (ab32381, 1:100, Abcam, Cambridge, MA, USA) at room temperature for 30 min. Beads in the negative control group were incubated with anti-IgG antibody. The centrifuge tubes containing the magnetic beads were placed on magnetic rack and the supernatant was removed, after which 500 μl RIP Wash Buffer was added and shaken by vortexing to remove the supernatant. The aforementioned procedures were repeated once. Then, 500 μl RIP Wash Buffer was added into the centrifuge tube. Afterwards, the tube was shaken by vortexing and placed on ice. The prepared tubes containing magnetic beads were placed on magnetic rack to remove the supernatant and then were added 900 μl RIP Immunoprecipitation Buffer. Prepared cell lysate was rapidly unfrozen and centrifuged at 14,000 rpm and 4℃ for 10 min. The supernatant of the cell lysate (100 μl) was added into bead-antibody complexes and cultured at 4℃ overnight. After transient centrifugation, the centrifuge tube was placed on magnetic rack to discard the supernatant. RIP Wash Buffer (500 μl) was added into the tube, shaken by vortexing and placed on magnetic rack to remove the supernatant, which was repeated for 6 times. Proteinase K Buffer (150 μl) was added into each tube to resuspend the bead-antibody complexes. Then, the mixture was cultured for 30 min at 55℃, after which the tube was placed on magnetic rack to pipette the supernatant. qRT-PCR was used to detect the expression of genes after RNA extraction.

### Dual luciferase reporter assay

Online software TargetScan (http://www.targetscan.org/vert_72/) was used to predict the binding sites of miR-424-5p in the 3’UTR of AOC1. Based on the results, wild and mutated sequences of the binding sites were separately synthesized and termed wt-AOC1 and mut-AOC1. Then, the sequences were inserted into luciferase reporter vectors (pGL3-Promoter, Promega, Madison, WI, USA) and cotransfected with miR-424-5p mimic (50 nM) or its negative control into HEK293T cells. The vectors containing wt-AOC1 or mut-AOC1 were also transfected with pRL-TK as the internal reference. After transfection, dual luciferase reporter gene detection kit (Promega, Madison, WI, USA) was used to assess firefly and renilla luciferase activities with renilla luciferase activity as the internal reference. Relative luciferase activity = firely luciferase activity/renilla luciferase activity.

### Microbubble preparation and cell treatment

Distearoyl phosphatidylcholine (5 mg), dipalmitonyl phosphatidyl ethanolamine (2 mg), plasmid (EGFP-miR-424-5p, 2 mg, Genepharma), Span 60 (1 mg) and glycerinum (50 μl) were added into a 1.5 ml tube and dissolved in PBS to make a final volume to 0.5 ml. Afterwards, the mixture were cultured at 37℃ for 30 min and then the tube was supplemented with perfluoropropane to replace air. The tube was shaken for 60 s and added 0.5 ml PBS before being placed for 5 min. Following disinfection, the tube was preserved at -80℃. When trophoblast cells HTR-8/Svneo and TEV-1 were treated with microbubbles, the mixture was exposed to ultrasound at 300 kHz and 0.5 W/cm^2^ for 30 s.

### MTT

HTR-8/Svneo and TEV-1 cells in logarithmic phase were digested by trypsin before being seeded onto 96 well plates (1000 cells/well, in triplicate). After being cultured at saturated humidity and 37℃ with 5% CO_2_ for 24 h, the cells were further cultured with 200 μl (1%, 5%, 10% or 20%) perfluoropropane microbubbles at the same condition. After cell culture, the supernatant was discarded and 10 μl DMSO-dissolved MTT (5 mg/ml, Sigma-Aldrich, Merck KGaA, Darmstadt, Germany) was mixed and cultured with the cells for 4 h at 37℃. Afterwards, the absorbance was detected at 570 nm (OD_570_). The absorbance was positively related to the number of living cells.

### qRT-PCR

RNA was isolated from placental tissues and trophoblast cells (HTR-8/Svneo and TEV-1) using TRIZOL reagent (Invitrogen, Carlsbad, CA, USA), and complementary DNA was obtained from RNA using reverse transcription kit (TaKaRa, Tokyo, Japan). RT-PCR amplifications were conducted based on the instructions of SYBR Green Mix kit (Roche Diagnostics, Indianapolis, IN) and the expression of genes of interest was determined on LightCycler 480 instrument (Roche, Indianapolis, IN, USA) by using U6 and GAPDH as the internal references of miRNA and mRNA, respectively. Thermal cycle parameters of RT-PCR amplifications were as follows: 95℃ for 10 s; 45 cycles of 95℃ for 5 s, 60℃ for 10 s and 72℃ for 10 s; extension at 72℃ for 5 min. Each PCR was performed in triplicates. Analysis of gene expression was conducted by 2^−ΔΔCt^ method. ΔΔCt = (Ct _target gene_ – Ct _internal reference_)_experimental group_ – (Ct _target gene_ – Ct _internal reference_)_control group_. Primer sequences of target genes and their internal references are exhibited in Table 1.

**Table 1:** Primer sequences of target genes and their internal references

Name of primer	Sequences
miR-424-5p-F	CAGCAGCAATTCATGT
miR-424-5p-R	TGGTGTCGTGGAGTCG
U6-F	CTCGCTTCGGCAGCACA
U6-R	AACGCTTCACGAATTTGCGT
AOC1-F	GAAGCAGAGCGAACTGGGAG
AOC1-R	TGGTACTTCTTGGGCAGCAG
β-catenin-F	CTGAGGAGCAGCTTCAGTCC
β-catenin-R	CCATCAAATCAGCTTGAGTAGCC
c-Myc-F	GCAATGCGTTGCTGGGTTAT
c-Myc-R	CGCATCCTTGTCCTGTGAGT
GAPDH-F	AATGGGCAGCCGTTAGGAAA
GAPDH-R	GCGCCCAATACGACCAAATC

*R* Reverse, *F* forward

### Western blotting

Placental tissues and trophoblast cells (HTR-8/Svneo and TEV-1) were lysed by RIPA lysis buffer (Beyotime, Shanghai, China) for protein extraction and the protein concentration was quantified by BCA kit (Beyotime, Shanghai, China). An equal volume of protein was mixed with loading buffer (Beyotime, Shanghai, China) and denatured through boiling water bath for 3 min. Then, electrophoresis was initiated at 80 V for 30 min and then switched to120 V for 1 ~ 2 h to separate the protein. Membrane transferring was carried out at 300 mA for 60 min through ice bath. Afterwards, the membrane was rinsed for 1 ~ 2 min and blocked at room temperature for 60 min or at 4℃ overnight. Primary antibodies against GAPDH (ab8245, 1:2000), AOC1 (ab231558, 1:1000), β-catenin (ab32572, 1:5000) and c-Myc (ab32072, 1:1000) (Abcam, Cambridge, MA, USA) were incubated with the membranes at room temperature for 1 h, respectively. Thereafter, the membranes were washed 3 × 10 min before being incubated with secondary antibody at room temperature for 1 h. Following washes 3 × 10 min, the membranes were subjected to color development. Chemiluminescence imaging system (Gel Doc XR, Bio-rad) was applied to visualize and quantify the protein bands.

### Animal experiments

Eight-week-old female C57BL/6 mice (25–30 g, Shanghai SLAC laboratory animal center, Shanghai, China) were reared under standard conditions (25 ± 5℃, 60–80% humidity, 12 h dark/light cycles, and standard diet) for one week of acclimation. Vaginal smears were daily obtained from female mice until the smears showed that they were oestrous. A PE mouse model was induced by subcutaneous injection of L-NAME [20].

Female mice were mated with male mice at a ratio of 2:1, and vaginal plug formation was detected on the next and defined as gestational day (GD) 0.5. The pregnant mice were divided into control, PE, and ultrasound microbubble groups (*n* = 6/group). During GD 7.5–18.5, the mice in PE and ultrasound microbubble groups were subcutaneously injected with 60 mg/kg L-NAME (Sigma-Aldrich, Merck KGaA, Darmstadt, Germany) every day, and mice in the control group were injected with an equal volume of normal saline. On GD 10.5, the mice in ultrasound microbubble group were injected with 100 μl microbubbles and irradiated by ultrasound at 1 MHz and 2 W/cm^2^ for 5 min with intervals of 10 s (on 10 s, off 10 s). The systolic pressure of the mice was measured on GD 6.5 and 18.5, and 24-h proteinuria was assayed using a BCA kit on GD 18.5. The mice were euthanatized on GD 18.5, and the placental tissues were collected for histological staining and gene expression detection.

### Hematoxylin and eosin (H&E) staining

The collected placental tissues were fixed in 4% paraformaldehyde solution and sliced into 4-μm paraffin sections. The sections were dewaxed in xylene, hydrated in alcohol gradient, stained with hematoxylin for 5 min, and immersed in 1% hydrochloric acid alcohol for 30 s. Following 2-min eosin staining, the sections were dehydrated, permeabilized, sealed, and observed by a microscopy.

### Statistical analysis

All data were analyzed by GraphPad prism 7 and summarized as mean ± standard deviation (SD). Normally distributed data between two groups were compared by *t*-test, and those among multiple groups were compared by one-way analysis of variance with Tukey’s multiple comparisons test. *P* value less than 0.05 was considered to have statistical significance.

## Results

### Downregulated miR-424-5p and upregulated AOC1 in the placentae of PE patients

**Fig. 1:**
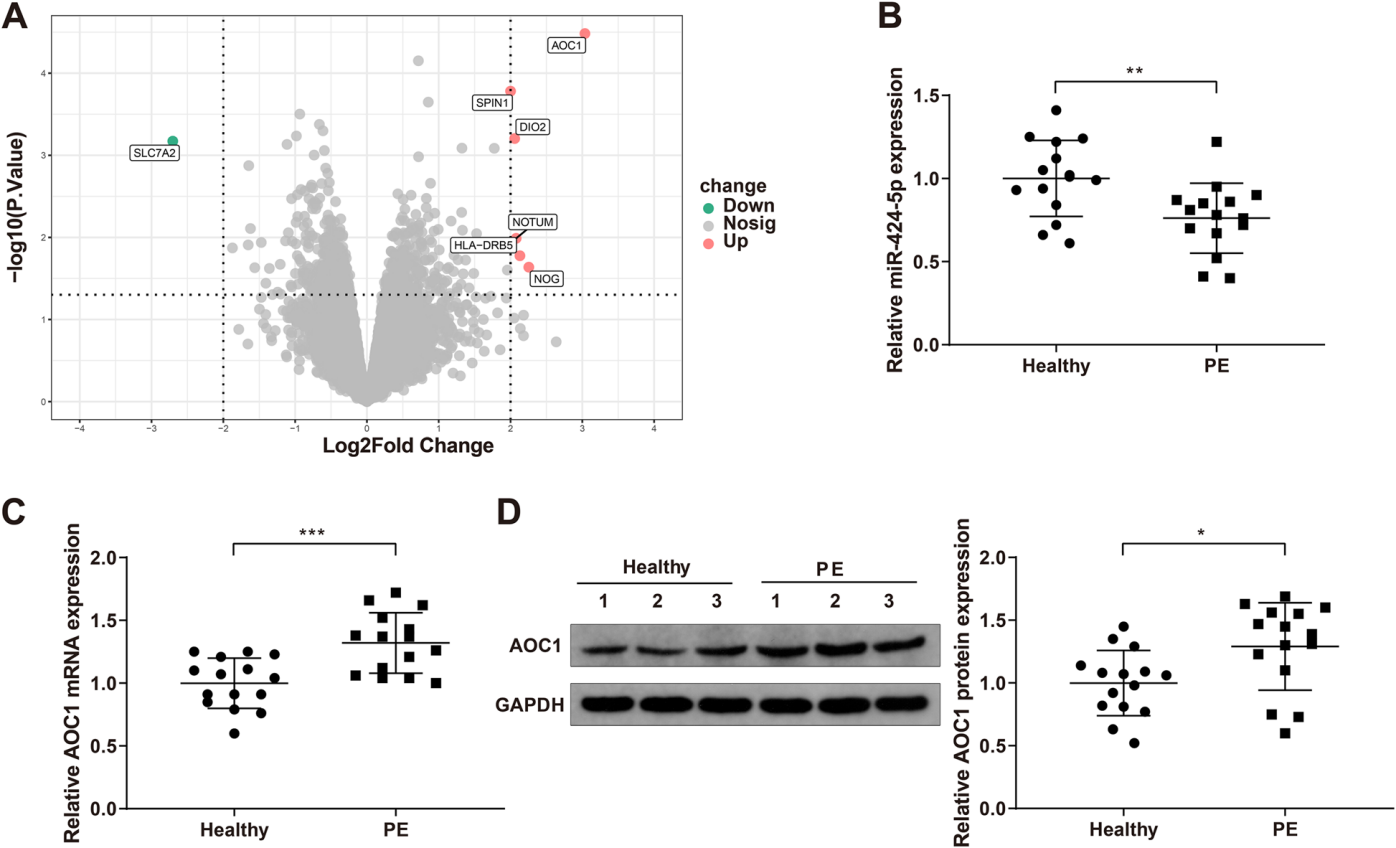
miR-424-5p was lowly expressed and AOC1 was highly expressed in the placentae of PE patients. Note: Based on bioinformatics analysis of GSE143953 data set, AOC1 was found to be upregulated in the placental tissues from PE patients (**A**); qRT-PCR quantified miR-424-5p (**B**) and AOC1 (**C**) expression in the placental tissues collected from pregnant women with PE and normal pregnant women; western blotting examined AOC1 protein expression in the collected placental tissues (**D**). * *P* < 0.05, ** *P* < 0.01, *** *P* < 0.001. PE, preeclampsia

Based on the data from PE patients and normal pregnant women, the age of the pregnant women between the two groups had no significant differences, while the systolic pressure, diastolic pressure and 24-h proteinuria of PE patients were much higher than those of the normal pregnant women (Table 2). Bioinformatics analysis of GSE143953 data set showed that AOC1 was overexpressed in the placental tissues of PE patients (Fig. 1A). qRT-PCR and western blot analyses detected miR-424-5p and AOC1 expression in the placental tissues. The results displayed a decrease of miR-424-5p expression in the placental tissues from PE patients (Fig. 1B, *P*< 0.01) as well as an increase of AOC1 expression (Fig. 1C-D, *P*< 0.05), compared to that in normal pregnant women. The results suggested that miR-424-5p and AOC1 might affect PE development.

**Table 2:** General data of PE patients and normal pregnant women

	Healthy control (*n* = 15)	PE (*n* = 15)	*P*
Age (y)	29.34 ± 2.68	31.27 ± 3.74	0.1155
Systolic pressure (mmHg)	112.34 ± 7.67	149.37 ± 9.82	< 0.001***
Diastolic pressure (mmHg)	76.34 ± 5.72	105.27 ± 7.16	< 0.001***
24-h proteinuria (g)	0	4.37 ± 0.38	< 0.001***

*PE* Preeclampsia

^***^P < 0.001

### miR-424-5p overexpression enhances the aggressive phenotype of trophoblast cells

**Fig. 2:**
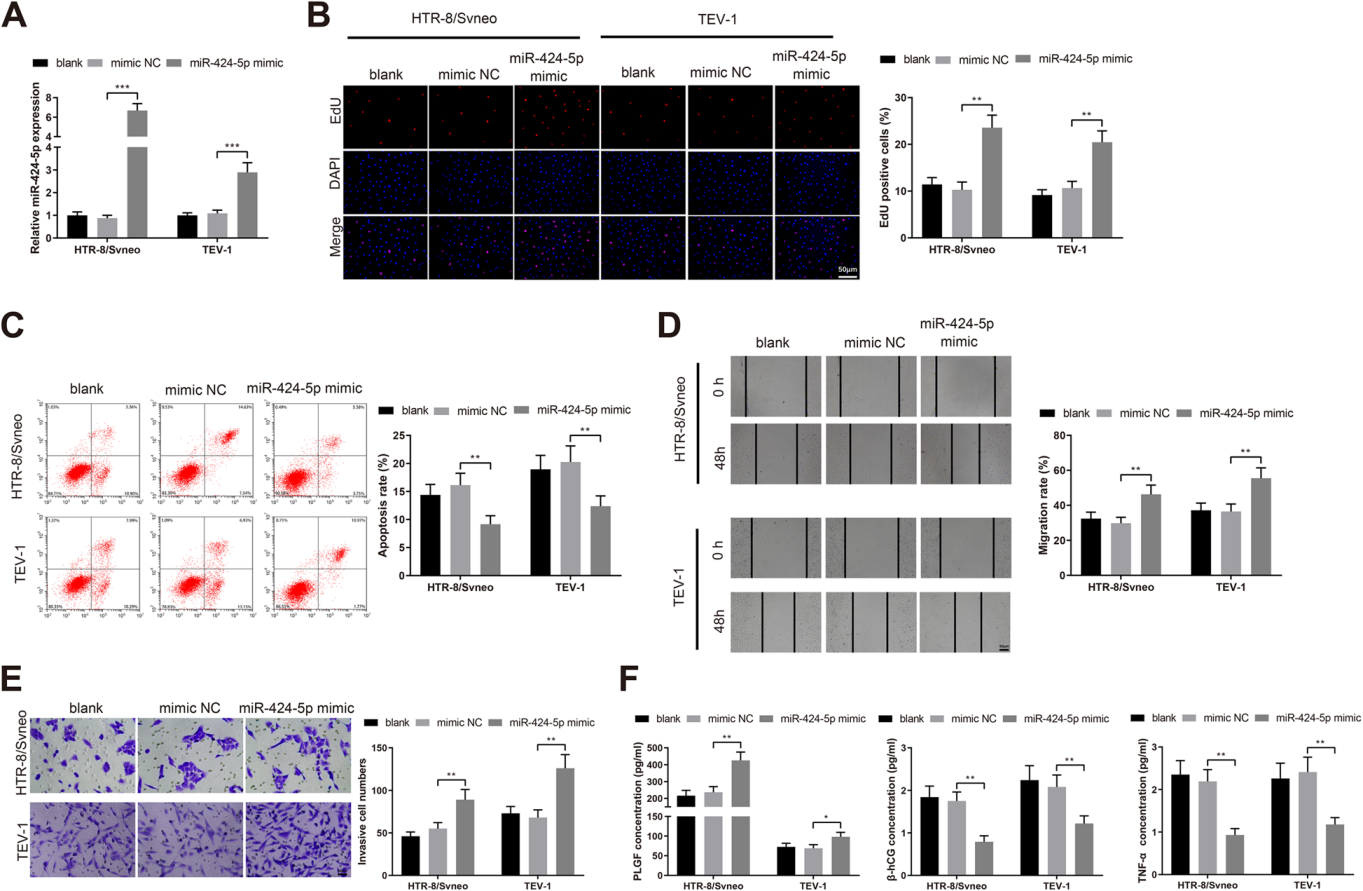
Overexpression of miR-424-5p promotes the proliferation, invasion and migration of trophoblast cells. Note: After transfection of miR-424-5p mimic or mimic NC into HTR-8/Svneo and TEV-1 cells, qRT-PCR determined the expression of miR-424-5p in HTR-8/Svneo and TEV-1 cells (**A**); trophoblast cell proliferation was detected through EdU staining (**B**); flow cytometry measured cell apoptosis rate (**C**); the migration and invasion capacities of trophoblast cells were assessed by Scratch assay (**D**) and Transwell invasion assay (**E**), respectively; the concentrations of PLGF, β-hCG and TNF-α in the supernatant were examined by ELISA (**F**). * *P* < 0.05, **P < 0.01, ***P < 0.001. EdU, 5-Ethynyl-2’-deoxyuridine; PLGF, placental growth factor; β-hCG, human chorionic gonadotropin; TNF-α, tumor necrosis factor-α

To investigate whether miR-424-5p could regulate the aggressive phenotype of trophoblast cells to participate in PE development, we transfected HTR-8/Svneo and TEV-1 cells with miR-424-5p mimic or mimic NC. qRT-PCR analysis of miR-424-5p expression exhibited an increment of miR-424-5p expression in the miR-424-5p mimic group compared to the mimic NC group (Fig. 2A, *P*< 0.001). As detected by EdU staining, HTR-8/Svneo and TEV-1 trophoblast cells transfected with miR-424-5p mimic had increased proliferation rate (Fig. 2B, *P*< 0.01 vs the mimic NC group). Consistently, flow cytometry revealed a decline in the apoptosis of HTR-8/Svneo and TEV-1 cells in the miR-424-5p mimic group compared to the mimic NC group (Fig. 2C, *P*< 0.01). Also, the migration rate and invasion rate of trophoblast cells (HTR-8/Svneo and TEV-1) in the miR-424-5p mimic group were promoted compared to the mimic NC group (Fig. 2D-E, *P*< 0.01). In addition, the level of PLGF in the supernatant of the HTR-8/Svneo and TEV-1 cell culture medium was upregulated, and β-hCG and TNF-α were downregulated in the miR-424-5p mimic group (Fig. 2F, *P*< 0.05, vs the mimic NC group). These findings indicated that miR-424-5p enhanced trophoblast cell growth, invasion and migration, indicating its role in PE pathogenesis.

### miR-424-5p targets AOC1

**Fig. 3:**
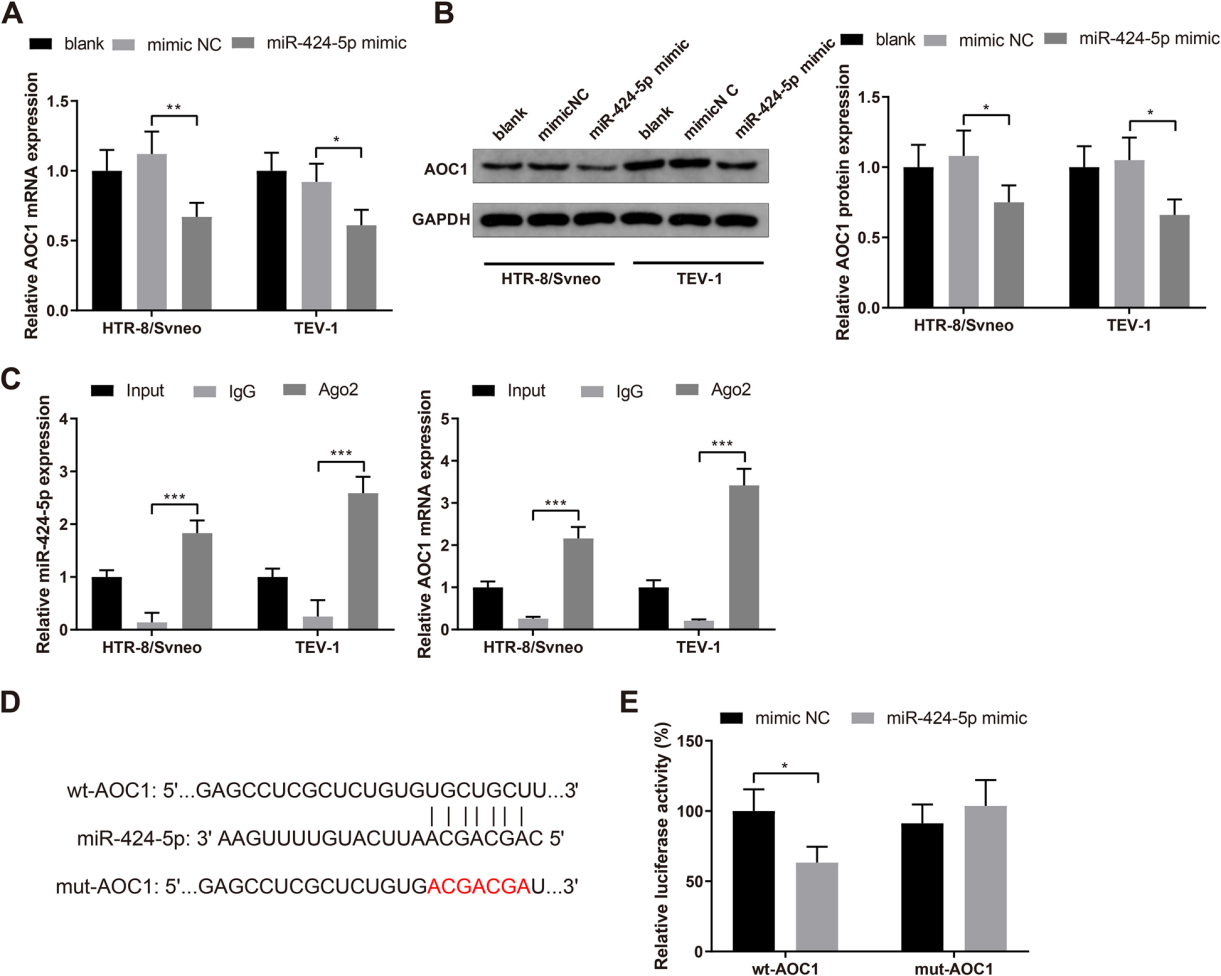
AOC1 is a target gene of miR-424-5p. Note: After HTR-8/Svneo and TEV-1 cells were transfected with miR-424-5p mimic or mimic NC, qRT-PCR (**A**) and western blotting (**B**) were used to detect AOC1 expression; the binding of miR-424-5p and AOC1 to the anti-Ago2 antibody or the negative control anti-IgG antibody in HTR-8/Svneo and TEV-1 trophoblast cells was shown by RIP assay (**C**); the binding sites of miR-424-5p in the 3’UTR of AOC1 were predicted by TargetScan (**D**); dual luciferase reporter assay identified the binding of miR-424-5p and AOC1 (**E**). * *P* < 0.05, **P < 0.01, ***P < 0.001. RIP, RNA immunoprecipitation; UTR, untranslated region; Ago2, argonaute-2; IgG, immunoglobulin G

After trophoblast cells HTR-8/Svneo and TEV-1 were transfected with miR-424-5p mimic or mimic NC, AOC1 expression was quantified by qRT-PCR and western blotting. The results described that transfection of miR-424-5p mimic potently suppressed AOC1 expression in HTR-8/Svneo and TEV-1 cells (Fig. 3A-B, *P*< 0.05). RIP assay revealed that anti Ago2 antibody significantly enriched AOC1 mRNA in HTR-8/Svneo and TEV-1 cells (Fig. 3C, *P*< 0.001). Dual luciferase reporter assay further identified the binding of miR-424-5p to AOC1. As predicted by TargetScan (http://www.targetscan.org/vert_72/), miR-424-5p had a binding sites in the 3’UTR of AOC1 (Fig. 3D). HEK293T cells cotransfected with the vectors containing wt-AOC1 and miR-424-5p mimic had reduced luciferase activity, while cotransfection of the vectors containing mut-AOC1 and miR-424-5p mimic did not affect the luciferase activity of HEK293T cells (Fig. 3E). Taken together, AOC1 is a target gene of miR-424-5p.

### miR-424-5p facilitates trophoblast invasiveness and migration by regulating AOC1

**Fig. 4:**
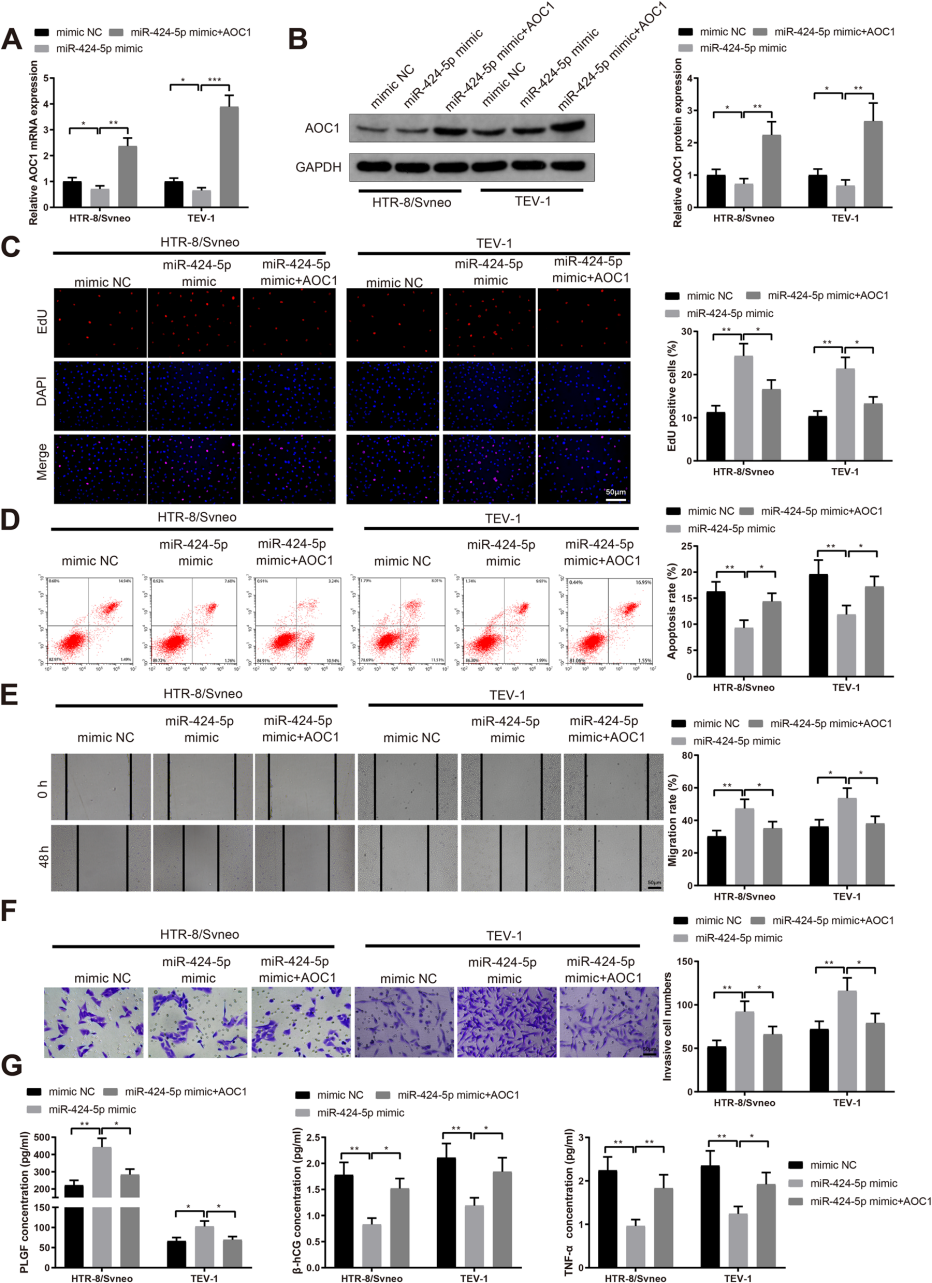
miR-424-5p enhances invasive and migratory behaviors of trophoblast cells through inhibiting AOC1. Note: After HTR-8/Svneo and TEV-1 cells were transfected with miR-424-5p mimic or miR-424-5p mimic + AOC1 overexpression vectors, AOC1 expression was determined by qRT-PCR (**A**) and western blotting (**B**); the proliferation rate of transfected cells was assessed by EdU staining (**C**) and the apoptosis rate by flow cytometry (**D**); Scratch assay detected the migration capacity of trophoblast cells (**E**); invasiveness of trophoblast cells were measured by Transwell invasion assay (**F**); ELISA examined the levels of PLGF, β-hCG and TNF-α in the supernatant (**G**). * *P* < 0.05, **P < 0.01, ***P < 0.001. EdU, 5-Ethynyl-2’-deoxyuridine; PLGF, placental growth factor; β-hCG, human chorionic gonadotropin; TNF-α, tumor necrosis factor-α

To identify whether miR-424-5p could affect trophoblast cell behaviors through regulating AOC1, we transfected trophoblast cells HTR-8/Svneo and TEV-1 with miR-424-5p mimic or miR-424-5p mimic plus AOC1 overexpression vectors. In the miR-424-5p mimic + AOC1 group, AOC1 expression was higher than that in the miR-424-5p mimic group (Fig. 4A-B, *P*< 0.05). The proliferation rate of HTR-8/Svneo and TEV-1 cells in the miR-424-5p mimic + AOC1 group was decreased (Fig. 4C, *P*< 0.05), and the apoptosis rate was promoted (Fig. 4D, *P*< 0.05) compared to that in the miR-424-5p mimic group. Detection of invasive and migratory behaviors of HTR-8/Svneo and TEV-1 cells exhibited decreases in the miR-424-5p mimic + AOC1 group (Fig. 4F, *P*< 0.05, vs the miR-424-5p mimic group). Consistently, the content of PLGF was downregulated after cotransfection of miR-424-5p mimic and AOC1 overexpression vectors, and the levels of β-hCG and TNF-α were upregulated in HTR-8/Svneo and TEV-1 cells (Fig. 4G, *P*< 0.05, vs the miR-424-5p mimic group). These results denoted that miR-424-5p regulated AOC1 expression to promote the proliferative, invasive and migratory capacities of HTR-8/Svneo and TEV-1 trophoblast cells, which might be associated with PE pathogenesis.

### Ultrasound/microbubble-mediated miR-424-5p delivery increases transfection efficiency

**Fig. 5:**
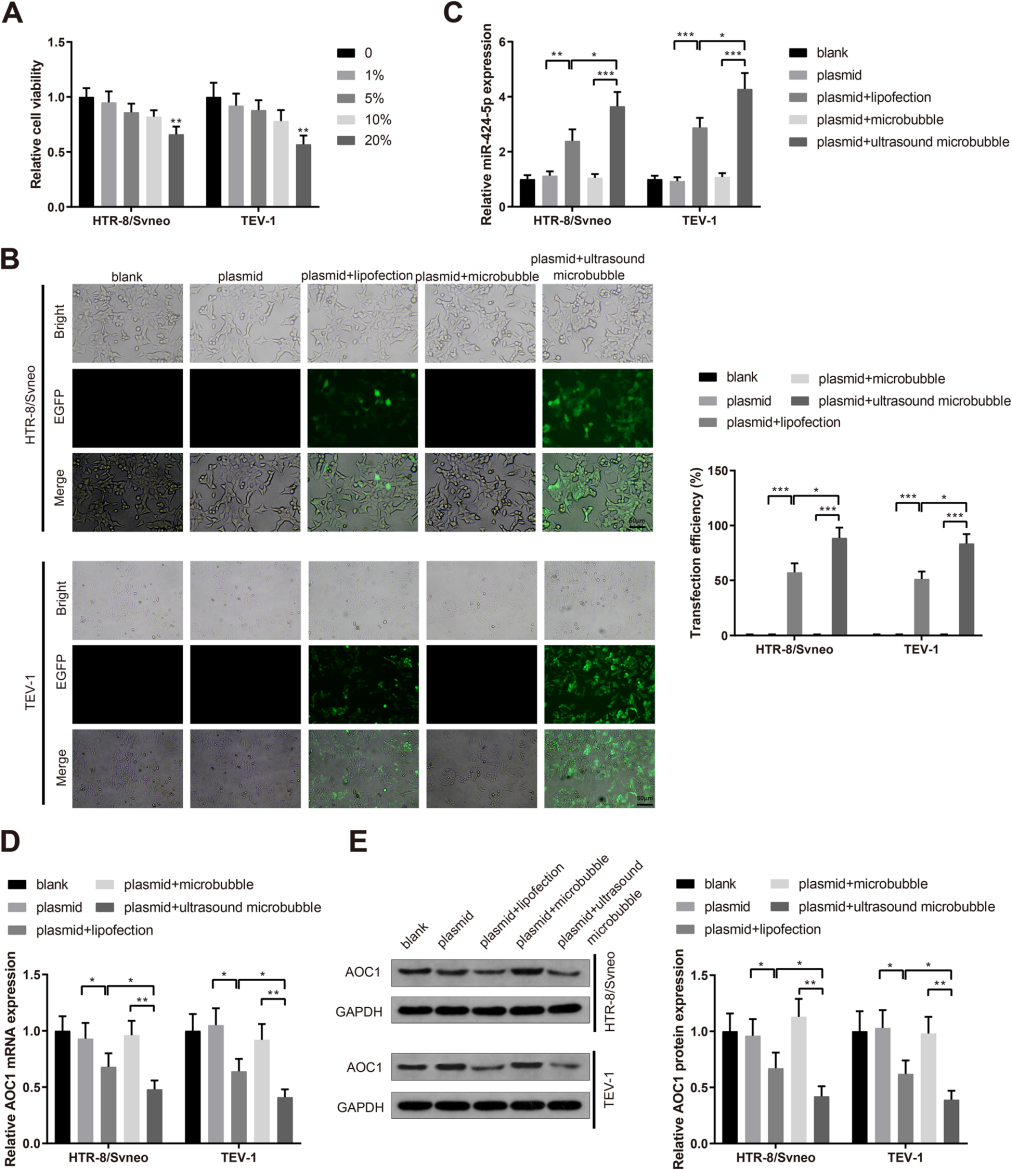
Ultrasound and microbubble mediated delivery significantly promotes transfection efficiency of miR-424-5p overexpression. Note: MTT assay determined the effects of perfluoropropane microbubbles with different concentrations on the viability of HTR-8/Svneo and TEV-1 trophoblast cells (**A**). After lipofection transfection and ultrasonic microbubble transfection, EGFP positive rate was measured to assess the transfection efficiency of miR-424-5p overexpression (**B**); qRT-PCR examined miR-424-5p (**C**) and AOC1 (**D**) expression levels; western blotting detected AOC1 protein expression levels (E). * *P* < 0.05, **P < 0.01, ***P < 0.001

The toxicity of perfluoropropane microbubbles on trophoblast cell viability was assayed by MTT. The results showed that the viability of HTR-8/Svneo and TEV-1 cells was declined when cultured with perfluoropropane microbubbles with the concentration of over 10% (Fig. 5A, *P*< 0.05), therefore, 10% perfluoropropane microbubbles was chosen for the following experiments. EGFP expression was assessed to evaluate the transfection efficiency. It was exhibited that EGFP-positive cells in the plasmid + ultrasound microbubble group was increased compared to those in the plasmid + microbubble group and the plasmid + lipofection group (Fig. 5B, *P*< 0.05). Moreover, miR-424-5p expression in HTR-8/Svneo and TEV-1 cells in the plasmid + ultrasound microbubble group was much higher (Fig. 5C, *P*< 0.05 and AOC1 expression was downregulated (Fig. 5D-E, *P*< 0.05) compared to those in the plasmid + microbubble group and the plasmid + lipofection group. Collectively, ultrasound and microbubble could promote the transfection efficiency of miR-424-5p delivery.

### Ultrasound and microbubble mediated miR-424-5p delivery promotes the aggressive phenotype of trophoblast cells

**Fig. 6:**
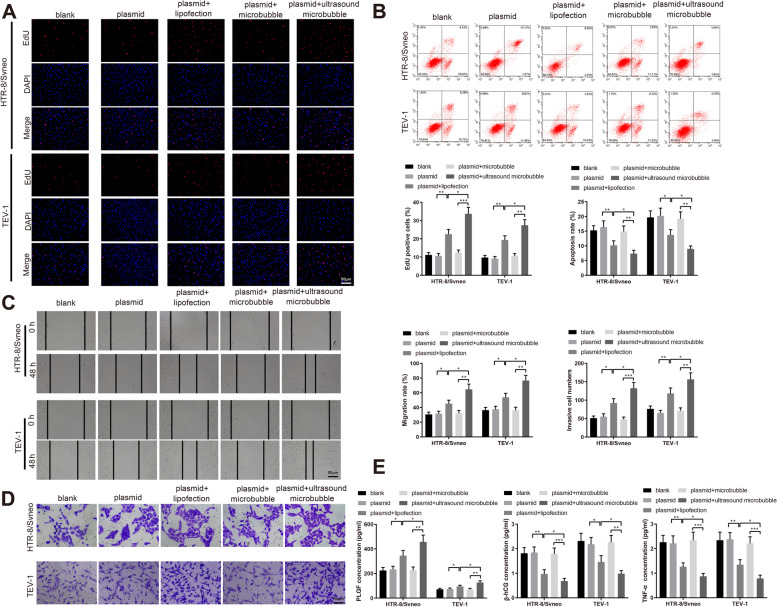
Ultrasound-mediated delivery of miR-424-5p loaded with microbubbles promotes the aggressive phenotype of trophoblast cells. Note: After transfection of lipofection and ultrasonic microbubbles with miR-424-5p overexpression, EdU staining was used to examine the proliferation rate of HTR-8/Svneo and TEV-1 trophoblast cells (**A**); apoptosis rate of HTR-8/Svneo and TEV-1 trophoblast cells were determined by flow cytometry (**B**), migration rate by Scratch assay (**C**) and invasiveness by Transwell invasion assay (**D**); ELISA examined the levels of PLGF, β-hCG and TNF-α in the supernatant (**E**). * *P* < 0.05, **P < 0.01, ***P < 0.001. EdU, 5-Ethynyl-2’-deoxyuridine; PLGF, placental growth factor; β-hCG, human chorionic gonadotropin; TNF-α, tumor necrosis factor-α

Next, we determined the effects of ultrasound-mediated delivery of miR-424-5p loaded with microbubbles on trophoblast cell invasive and migratory behaviors. HTR-8/Svneo and TEV-1 cells subjected to ultrasonic microbubble carried miR-424-5p transfection showed increased proliferation rate, decreased apoptosis rate and prompted migration and invasion rates compared to those in the plasmid + lipofection group and the plasmid + microbubble group (Fig. 6A-D, *P*< 0.05). Also, the concentration of PLGF was upregulated and the levels of β-hCG and TNF-α were downregulated in HTR-8/Svneo and TEV-1 cells in the plasmid + ultrasound microbubble group (Fig. 6E, *P*< 0.05, vs the plasmid + lipofection group or the plasmid + microbubble group). As suggested by these results, ultrasound and microbubble mediated miR-424-5p delivery could potently facilitate the invasive and migratory behaviors of HTR-8/Svneo and TEV-1 trophoblast cells.

### miR-424-5p targets AOC1 to activate Wnt/β-catenin signaling pathway in trophoblast cells

**Fig. 7:**
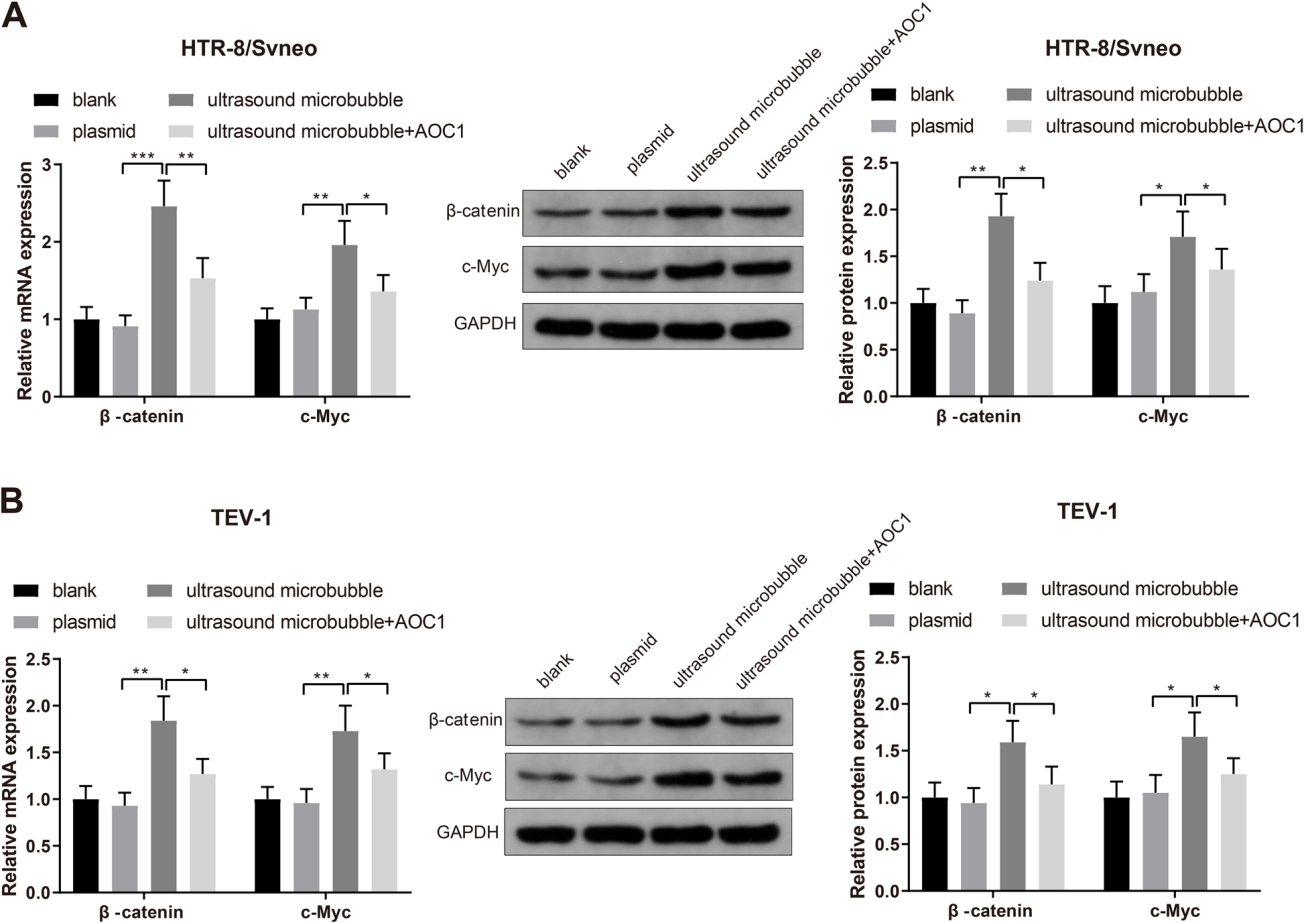
miR-424-5p activates Wnt/β-catenin signaling pathway through targeting AOC1. Note: After transfection of miR-424-5p overexpression or miR-424-5p overexpression plus AOC1 overexpression in HTR-8/Svneo and TEV-1 cells, the expression levels of β-catenin and c-Myc were quantified by qRT-PCR and western blotting. * *P* < 0.05, **P < 0.01, ***P < 0.001

HTR-8/Svneo and TEV-1 trophoblast cells were transfected with miR-424-5p overexpression plasmid or miR-424-5p overexpression plasmid plus AOC1 overexpression plasmid (lipofection transfection) by using ultrasonic microbubble transfection to evaluate the activation of Wnt/β-catenin signaling pathway. qRT-PCR and western blot analyses displayed that the expression levels of β-catenin and c-Myc was upregulated in the ultrasound microbubble group compared to those in the plasmid group and the ultrasound microbubble + AOC1 group (Fig. 7A-B, *P*< 0.05). Therefore, it was confirmed that miR-424-5p targeted AOC1 to activate Wnt/β-catenin signaling pathway in trophoblast cells.

### Ultrasound/microbubble-mediated miR-424-5p delivery attenuates PE development in mice

**Fig. 8:**
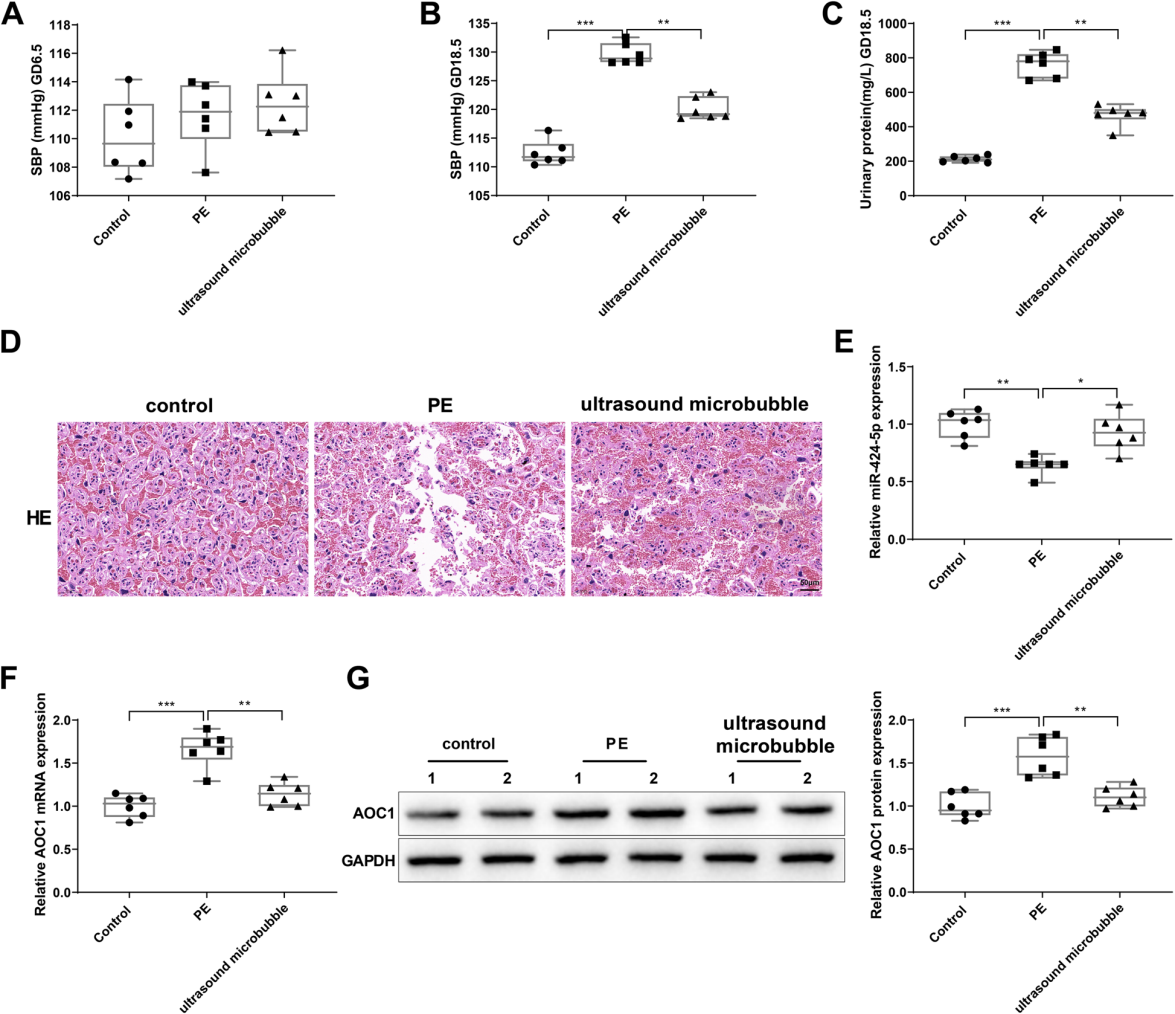
Ultrasound/microbubble-mediated miR-424-5p delivery blocks PE development in mice. Note: Mice were subcutaneously injected with L-NAME and treated with ultrasound/microbubbles loaded with miR-424-5p overexpression plasmids. The systolic pressure of mice was evaluated on GD 6.5 (**A**) and GD 18.5 (**B**); the proteinuria of mice was detected on GD 18.5 (**C**); the histopathological changes in mouse placental tissues were evaluated by hematoxylin and eosin staining (**D**); qRT-PCR was used to detect miR-424-5p expression in mouse placental tissues (**E**); qRT-PCR and western blot were used to measure AOC1 mRNA (**F**) and protein (**G**) levels in mouse placentae. *N* = 6, * *P* < 0.05, **P < 0.01, ***P < 0.001. PE, preeclampsia; GD, gestational day

The effect of ultrasound/microbubble-mediated miR-424-5p delivery in PE was verified in a mouse model. PE is characterized by an increase in systolic pressure and existence of proteinuria. On GD 6.5, the systolic pressure of mice in the three groups had no significant difference (Fig. 8A). On GD 18.5, the systolic pressure of mice in PE group was higher than that in control group, and mice in ultrasound microbubble group had lower systolic pressure than mice in PE group (Fig. 8B, *P*< 0.05). Compared to control group, PE group showed increased proteinuria on GD18.5, but ultrasound microbubble group had decreased proteinuria on GD18.5 than PE group (Fig. 8C, *P*< 0.05). Moreover, the placental tissues of PE mice showed fibrinoid necrosis and less villous vessels, and ultrasound and microbubble treatment significantly reduced the histopathological changes in PE mice (Fig. 8D). qRT-PCR and western blot results suggested that miR-424-5p expressed at a low level and AOC1 expressed at a high level in the placental tissues of PE mice, and ultrasound and microbubble treatment resulted in increased miR-424-5p expression and decreased AOC1 expression (Fig. 8E-G, *P*< 0.05). Overall, ultrasound/microbubble-mediated miR-424-5p delivery showed a therapeutic effect on experimental-induced PE in mice.

## Discussion

This study demonstrated downregulated miR-424-5p and upregulated AOC1 in placental tissues from PE patients. Specific gene delivery to trophoblast cells by ultrasound exposure and microbubbles increased miR-424-5p expression in trophoblast cells. Moreover, our findings indicated that miR-424-5p overexpression enhances trophoblast cell migration and invasion through inhibiting AOC1 and activating Wnt/β-catenin signaling pathway. Our research developed an explicit understanding of the role of miR-424-5p in trophoblast cell invasion and migration and presented a promising prospect of ultrasonic microbubble as a gene delivery tool in trophoblast cells.

miR-424-5p is confirmed as a tumor suppressor in ovarian cancer [21] and cervical cancer [22], which is able to inhibit malignant behaviors of tumors through targeting different mRNAs. In esophageal squamous cell carcinoma, miR-424-5p overexpression provokes the propagation and migration of cancer cells [23]. Mouillet et al. has demonstrated that miR-424 expression was uniquely reduced in primary human trophoblasts upon exposure to hypoxia in contrast to that in non-trophoblastic cell types, which might be attributed to the reduction of trophoblast cell differentiation [14]. In PE placental samples, abnormal miR-424 expression in women with pregnancy-related hypertension is reported to affect the activity of Wnt signaling pathway [24]. In this study, miR-424-5p was downregulated in the placental tissues from PE patients. The overexpression of miR-424-5p promoted the proliferation of trophoblast cells as well as the invasive and migratory capacities while suppressing cell apoptosis. To further investigate the mechanisms by which miR-424-5p regulated trophoblast cell development, we then searched for the possible target genes of miR-424-5p in regulating trophoblast cell behaviors. AOC1 is reported as a secreted diamine oxidase responsible for deamination of putrescine and histamine and generation of hydrogen peroxide, which is overexpressed in the kidneys, placenta, intestine and lungs [25]. Based on the bioinformatics analysis of GSE143953 data set, AOC1 was demonstrated to be upregulated in PE placental tissues. Moreover, there was a predicted binding site for miR-424-5p in the 3’UTR of AOC1. Through RIP assay and dual luciferase reporter assay, we identified that miR-424-5p could bind to AOC1. AOC1 knockdown in gastric cancer cells could suppress the propagation, invasiveness and migration, induce activation of the caspase cascade, and inactivated AKT signaling pathway [26]. A previous investigation also reported that miR-424 targeted FGFR1 in trophoblast cells and controlled FGFR1 expression in altered differentiation or under hypoxic condition [14]. This study found that AOC1 was downregulated by miR-424-5p and the overexpression of AOC1 significantly inhibited miR-424-5p-mediated enhancement of trophoblast cell propagation, invasion and migration. However, it should be cautious that, due to the absence of control of the level of transfection in the cellular models, this artificial approach can give indications about the link with preeclampsia but only indirectly.

Our results indicated that miR-424-5p upregulation in trophoblast cells might be an effective strategy for PE treatment through promoting the acquisition of trophoblast cell migration and invasion. However, transfection efficiency is a major challenge for clinical application. In recent years, lentiviral vector is widely used as the carrier for gene expression regulation in trophoblast cells [27, 28]. Adenoviral vector has been used to deliver VEGF_121_ to BPH/5 mice via tail vein and the authors confirmed that adenoviral-mediated delivery of VEGF_121_ upregulated the plasma-free VEGF level, accompanied by restored angiogenic ability, reduced fetal resorptions and prevented hypertension and proteinuria at the late gestation [29]. Compared with viral vectors which have high transfection efficiency but poor safety and organ specificity [30], ultrasound and microbubble mediated gene delivery, as a non-viral method, is a safe, effective and targeted approach for gene transfection [31, 32]. Cui et al. has utilized ultrasonic microbubble technique to transfer integrin-linked kinase (ILK) into endothelial progenitor cells, and the study demonstrated that ultrasound and microbubble mediated ILK delivery promoted angiogenic properties of endothelial progenitor cells and thus inhibited the development of PE [31]. This study utilizing ultrasonic microbubble to deliver miR-424-5p into trophoblast cells successfully improved the transfection efficiency. Ultrasound and microbubble mediated miR-424-5p delivery further promoted the effects of miR-424-5p on trophoblast cell development and also showed a therapeutic effect in experimental PE in mice. Activated Wnt/β-catenin signaling pathway could enhance the migration and invasiveness of trophoblast cells and inhibit PE development [33, 34]. Considering the potential regulation of miR-424-5p on Wnt signaling pathway, we detected the activation of Wnt/β-catenin signaling pathway. After transfection of miR-424-5p carried by ultrasonic microbubbles into trophoblast cells, we found increased levels of β-catenin and c-Myc, and the overexpression of AOC1 decreased β-catenin and c-Myc expression levels. Therefore, we proposed that miR-424-5p targeted AOC1 and then activated Wnt/β-catenin signaling pathway.

## Conclusion

In this paper, we confirmed that miR-424-5p was decreased in PE placental tissues and miR-424-5p targeted and suppressed AOC1 in trophoblast cells. miR-424-5p enhanced the aggressive phenotype of trophoblast cells. We also provided evidence that ultrasound microbubble-mediated miR-424-5p delivery promoted transfection efficiency in trophoblast cells and further facilitated the promotive effects of miR-424-5p on the migratory and invasive capacities of trophoblast cells. Our study reveals a novel miR-424-5p-mediated mechanism modulating aggressive phenotype of trophoblast cells and might provide a novel insight for PE treatment.

## Data Availability

The datasets used or analyzed during the current study are available from the corresponding author on reasonable request.

## Abbreviations

PE: Preeclampsia
PLGF: Placental growth factor
TNF-α: Tumor necrosis factor-α
RIP: RNA immunoprecipitation
UTR: Untranslated region
miRNAs: MicroRNAs
FBS: Fetal bovine serum
EdU: Ethynyl-2’-deoxyuridine
SD: Standard deviation
